# Functional Hydrogels for Agricultural Application

**DOI:** 10.3390/gels9070590

**Published:** 2023-07-22

**Authors:** Romana Kratochvílová, Milan Kráčalík, Marcela Smilková, Petr Sedláček, Miloslav Pekař, Elke Bradt, Jiří Smilek, Petra Závodská, Martina Klučáková

**Affiliations:** 1 Faculty of Chemistry, Brno University of Technology, Purkyňova 464/118, CZ-61200 Brno, Czech Republic; kolajova@fch.vutbr.cz (R.K.); smilkova@fch.vut.cz (M.S.); sedlacek-p@fch.vut.cz (P.S.); pekar@fch.vut.cz (M.P.); smilek@fch.vut.cz (J.S.); petra.zavodska@vut.cz (P.Z.); 2 Institute of Polymer Science, Johannes Kepler University, Altenberger Strasse 69, 4040 Linz, Austria; milan.kracalik@jku.at (M.K.); elke.bradt@jku.at (E.B.)

**Keywords:** superabsorbent, swelling, fertilizer, lignohumate, rheology, stability

## Abstract

Ten different hydrogels were prepared and analyzed from the point of view of their use in soil. FT-IR spectra, morphology, swelling ability, and rheological properties were determined for their characterization and appraisal of their stability. The aim was to characterize prepared materials containing different amounts of NPK as mineral fertilizer, lignohumate as a source of organic carbon, and its combination. This study of stability was focused on utility properties in their application in soil—repeated drying/re-swelling cycles and possible freezing in winter. Lignohumate supported the water absorbency, while the addition of NPK caused a negative effect. Pore sizes decreased with NPK addition. Lignohumate incorporated into polymers resulted in a much miscellaneous structure, rich in different pores and voids of with a wide range of sizes. NPK fertilizer supported the elastic character of prepared materials, while the addition of lignohumate shifted their rheological behavior to more liquid. Both dynamic moduli decreased in time. The most stable samples appeared to contain only one fertilizer constituent (NPK or lignohumate). Repeated re-swelling resulted in an increase in elastic character, which was connected with the gradual release of fertilizers. A similar effect was observed with samples that were frozen and defrosted, except samples containing a higher amount of NPK without lignohumate. A positive effect of acrylamide on superabsorbent properties was not confirmed.

## 1. Introduction

Superabsorbent polymers are hydrogels created by freely crosslinked three-dimensional networks of flexible polymeric chains homogenously distributed in a water dispersion medium. From a structural point of view, they are crosslinked polyelectrolytes [1]. They have the unique ability to absorb and retain relatively large amounts of water or water solutions in their structure as a result of rising entropy inside the polymer chain network [1,2,3,4,5,6]. Their field of application is very wide. They are suitable where the absorption of liquids is required. The most common application is in sanitary equipment. They are very often used as targeted carriers of special substances, e.g., in agriculture, to ensure sufficient humidity for cultivated crops [7,8,9,10,11,12].

Inspired by published works [2,3,7,8,9,10,11,12] and their results, the composite materials for the controlled release of mineral nutrients and humic substances for agricultural application were developed in our previous work [13]. In this work, we are focused on the characterization of prepared materials by means of FT-IR, SEM, and rheology. The aim of this work is to investigate materials from the point of view of their agricultural application. It means that the effect of freezing and repeated re-swelling on the mechanical properties of storage and loss moduli was investigated.

In the beginning, we investigated results available in studies published for superabsorbent polymers with similar compositions. Li et al. [8] synthetized a poly(acrylic acid)/sodium humate superabsorbent composite and studied its water absorbency properties. The effects of the initial concentration of acrylic acid, its neutralization degree, contents of crosslinker (*N*,*N′*-methylenebisacrylamide), initiator (ammonium persulfate), and sodium humate as an active filler were investigated. It was found that the water absorbency was much higher in distilled water compared with the NaCl solution. The addition of sodium humate supported material swelling up to 20% wt. A decrease in swelling was observed for higher additions. Water absorbency gradually decreased with the re-swelling cycles up to ~70% wt. of the initial value for the fifth cycle. Materials were characterized by FT-IR spectrometry and thermogravimetry. Similar studies were realized by Chu et al. [9,10]. They synthetized superabsorbents based on the same starting materials, but their achieved water absorbency was much lower [9]. They studied swelling in various saline solutions and observed a linear relationship between the saturated water absorbency and the minus square root of the ionic strength of the external medium. The water absorbency of PAA-AM/KHA in various salt solutions had the following order: NH_4_Cl_(aq)_ = KCl_(aq)_ = NaCl_(aq)_ > MgCl_2(aq)_ > CaCl_2(aq)_ > AlCl_3(aq)_ > FeCl_3(aq)_ [10]. Liu et al. [11] prepared a multifunctional superabsorbent based on chitosan, g-poly(acrylic acid), and sodium humate. They stated that sodium humate enhanced water absorbency, and the content of 10 wt% sodium humate gave the best absorption. Gao et al. [12] investigated the effects of N, reaction temperature, and contents of initial materials on water absorption. They confirmed the positive effect of the addition of humic acid on water absorbency. 

Smart hydrogels for agricultural applications were recently developed and investigated [14,15]. Obtained results and characteristics were described in several reviews [16,17,18,19]. Zhang et al. [14] developed new multifunctional smart soils with double-layer structures consisting of zwitterionic, thermo-responsive poly(NIPAM-*co*-VPES), and poly(NIPAM-*co*-SBAA) aerogels doped in the upper layer and the bottom bed of the soils, respectively. Pushpamalar et al. [15] developed eco-friendly smart hydrogels for soil conditioning and sustain release fertilizer generated from biomass waste. A review focused on cellulose-based hydrogel materials and their prospective applications in agricultural activity was published by Kabir et al. [16]. Another review encompassed the latest developments in the field of smart hydrogel synthesis based on their unique features and different aspects of their responsive behaviors was published by Sikdar et al. [17]. Azeem et al. [18] published the review that spotlighted application prospects of three-dimensional hydrogel in agriculture. Hydrogel functioning, significance, advantages, mechanism of fertilizer release, and agriculture-specific applications were comprehensively described. In the review of Chakraborty et al. [19], the use of different nanocomposite materials developed for nutrient management in agriculture was summarized with a major focus on their synthesis and characterization techniques and application aspects in plant nutrition, along with addressing constraints and future opportunities of this domain.

Effects of structural variables on water absorbency and rheological behavior of superabsorbents based on acrylic polymers were investigated by Ramazani-Harandi et al. [20]. Their results showed a linear correlation between water absorbency and storage modulus over the rubber-elastic plateau. The swelling improvement and swollen gel strength were observed with increased crosslinker concentration. Zaharia et al. [21] enriched a poly(acrylic acid-co-N,N′-methylene-bis-acrylamide) composite hydrogel with bacterial cellulose. Infrared spectra, thermogravimetric curve, XRD diffraction pattern, SEM micrograph, swelling degree, and rheological properties were determined and investigated for both basic polymer and enriched material. These results can be used for the comparison with our experimental data. A review focused on the rheological properties and behavior of polymer hydrogels for different industrial applications was published by Rehman and Shah [22].

NPK, as a mineral nutrient used in agriculture, is often combined with different organic manures in order to achieve suitable fertilization for soil health, plant growth, and crop yields [23,24,25]. In this work, the amendment of manure was replaced by lignohumate as the source of organic carbon. It is an industrially produced analog of natural humic substances produced by the thermal processing of technical lignosulfonate, which is based on the oxidation and hydrolytic destruction of lignin-containing raw materials [26,27,28,29]. Due to its smaller molecular size and weight in comparison to humic substances isolated from native sources, it is more soluble and has a similar character to the most active fractions of humic substances dissolved in soil solution [30,31,32,33]. The characterization of lignohumate using elemental analysis and spectral methods and its comparison with humic substances isolated from different raw materials can be found in refs. [27,34]. The unique properties and supramolecular character of lignohumate can result in the promotion of plant growth connected with its utilization for agricultural and horticultural purposes [27,35,36,37,38,39,40].

The above-mentioned references provide characteristics for the comparison with functional materials investigated in this study. Our materials are based on poly(acrylic acid) (AA). *N,N*´-methylenebisacrylamide (MBA) was used as a crosslinker, and potassium peroxydisulfate (KPS) as an initiator. The polymer was enriched by potassium lignohumate (LH) as a source of organic carbon and NPK as a mineral nutrient [13]. The effect of their additions on the properties of prepared functional materials was investigated. In this work, the addition of acrylamide (AM), which was used in several references [2,3,5,10], was also studied in order to consider its influence on the properties of superabsorbent materials. However, the use of acrylamide is problematic because of its toxicity [41,42,43,44]; therefore, the superabsorbent polymers containing higher amounts of acrylamide are not friendly for agricultural use.

## 2. Results and Discussion

Ten different superabsorbent materials were prepared in order to investigate the effect of inorganic and organic nutrients, as well as acrylamide, on the material properties. The composition of prepared materials is described in Table 1. AA, KOH, MBA, and KPS were used for the preparation of all superabsorbent materials in the same amounts; therefore, only constituents differed in individual samples are listed in Table 1. Samples A…F contained mineral nutrient NPK in lower (A, C, E) and higher (B, D, F) amounts. Samples C…F combined NPK with LH, and samples G and H contained only LH. Samples I and J were without inorganic and organic nutrients and were analyzed for comparison with others. Alternatively, materials containing acrylamide (E, F, G, J) were prepared for the comparison of their properties and behavior. In consideration of AM toxicity, its content was relatively low. The AM/AA ratio was 5/95 in comparison with [45], where the ratios were from 30/70 to 70/30.

### 2.1. FT-IR Spectrometry

**Figure 1:**
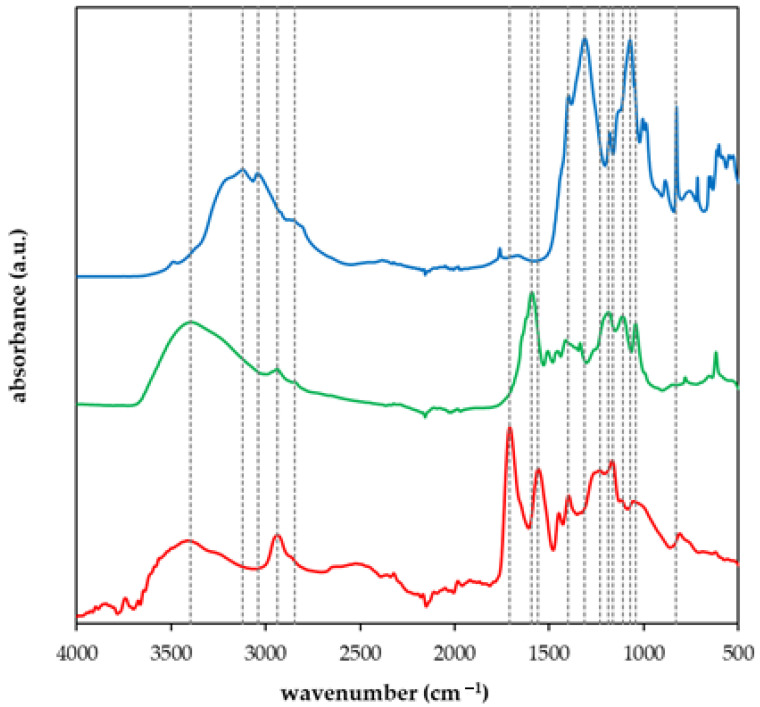
FT-IR spectra of NPK (blue), lignohumate (green), and sample A (red). Dotted lines are added for easy comparison of peaks in spectra.

FT-IR spectra of prepared materials, NPK, and lignohumate were collected. Samples were measured in dry forms by means of the ATR technique. In Figure 1, spectra for NPK, LH, and sample I are compared. Spectra were collected for the comparison of different prepared superabsorbent polymers and the identification of their constituents. The spectrum of NPK was described in detail in [45,46,47]. Peaks between 3000 and 3500 cm^−1^ can be assigned to stretching modes of O-H and N-H bonds in urea, potassium dihydrogen phosphate, and ammonium dihydrogen phosphate. The shoulder around 2800 cm^−1^ corresponds to the O-H stretching. Peaks between 1300 and 1400 cm^−1^ belong to P=O stretching, 1160 cm^−1^ is related to P-OH stretching, and 1070 cm^−1^ is attributed to the HO-P-OH bending. The O-N=P and O-N bonds in ammonium dihydrogen phosphate appear as peaks between 700 and 800 cm^−1^ [45,46]. The spectrum of lignohumate was analyzed and compared with natural humic substances in ref. [29]. The broad peak above 3000 cm^−1^ belongs to O-H stretching and N–H stretching in different functional groups and overlaps vibrations of C-H groups in aromatic structures. Bands between 2800 and 3000 cm^−1^ can be assigned to C–H and CH_2_ groups. Aromatic C=C vibrations and C=O stretching of quinone and amide groups can be observed between 1600 and 1640 cm^−1^. N–H deformation and C=N stretching of amides appear between 1500 and 1520 cm^−1^. Bands between 1400 and 1500 cm^−1^ can be assigned to C-H bending of the CH_3_ group, O-H deformation, and C-O stretching phenolic groups. C=O stretching of aryl esters and C-O stretching of aryl ethers and phenols can be seen between 1200 and 1300 cm^−1^. C-O stretching of secondary alcohols, ethers, and polysaccharides or polysaccharide-like substances appear between 1000 and 1200 cm^−1^ [34,48]. Superabsorbent polymers based on AA and AM, as well as the starting materials, were characterized in ref. [49]. Peaks between 3000 and 3500 cm^−1^ belong to O-H and N-H stretching. The peak of C-H in the methylene group is visible at 2940 cm^−1^. Two bands between 1550 and 1720 cm^−1^ can be assigned to C=C and C-O vibrations. C-O vibrations also appear between 1100 and 1200 cm^−1^.

**Figure 2:**
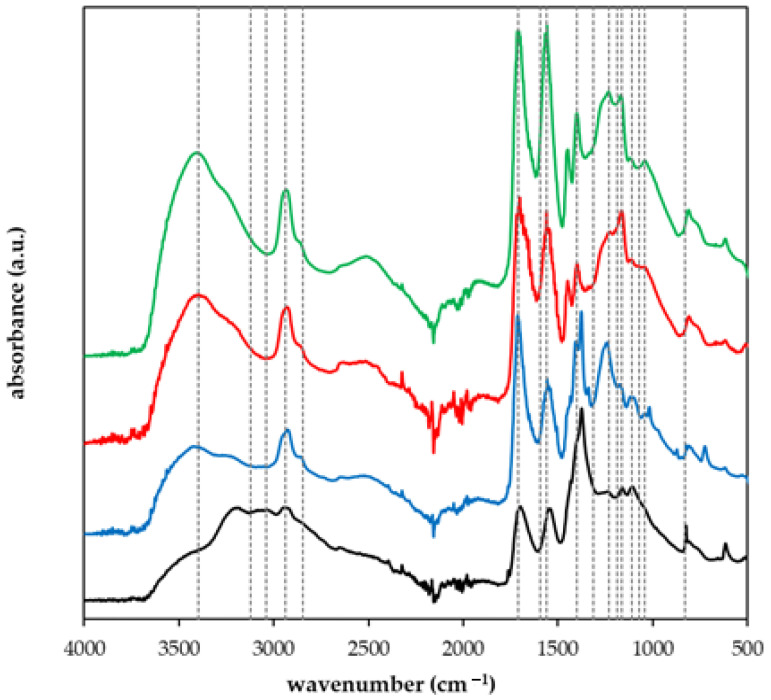
FT-IR spectra of samples C (blue), D (black), G (red), and H (green). Dotted lines are added for easy comparison of peaks in spectra.

FT-IR spectra of some prepared hydrogels are shown in Figure 2. We can see that the presence of NPK fertilizer came through mainly in the spectrum of sample D which contains ten times higher amounts in comparison with sample C. Characteristic peaks observable at 1390 cm^−1^, 1140 cm^−1^, 1160 cm^−1^, and 1100 cm^−1^ are related to P=O stretching, P-OH stretching, and 1070 cm^−1^, and HO-P-OH bending, respectively. The peaks shifted slightly in comparison with the spectrum of NPK (Figure 1). Samples G and H are without NPK and contain LH as a source of organic carbon. The difference between the two samples is the addition of AM (sample G). No noticeable differences in the spectra of both samples were observed.

### 2.2. SEM Analysis

**Figure 3:**
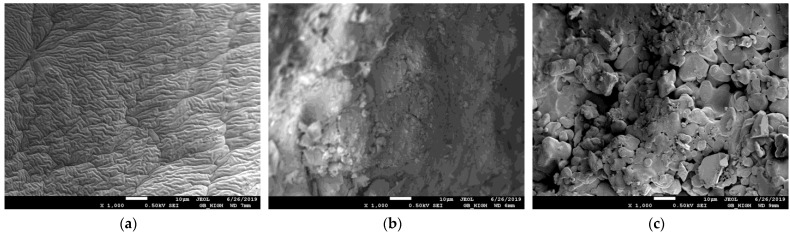
SEM photos of samples A with low content of NPK (**a**), B with high content of NPK (**b**), and D with high content of NPK and LH (**c**) before swelling.

The morphology of superabsorbent polymers was characterized by means of scanning electron microscopy (SEM). At first, the prepared samples before swelling were analyzed in order to discover possible changes in their surface given by different constituents added to the basic polymer constituents. In Figure 3, the photos of samples with low and high contents of NPK and the effect of LH addition are shown.

As can be seen, the surface of sample A has a wrinkly character without visible pores and voids (Figure 3a). The surface morphology changed if the content of NPK increased (ten times). The crystals of NPK constituents (urea, potassium dihydrogen phosphate, and ammonium dihydrogen phosphate) are visible on the surface, which became more heterogeneous (Figure 3b). A similar effect on the surface morphology of superabsorbent polymers (if inorganic nutrients were incorporated in them) was observed in refs. [45,50,51].

The deposition of NPK fertilizer and the crystals of their constituents deposited onto pores walls were also observed in the swollen state in the higher magnification [45,51]. The surface roughness also increased after LH addition (Figure 3c) which can suppose the swelling ability of the superabsorbent (see Section 2.3.). The positive effect of humic substances on the formation of the porous structure was also observed in refs. [9,12,52].

**Figure 4:**
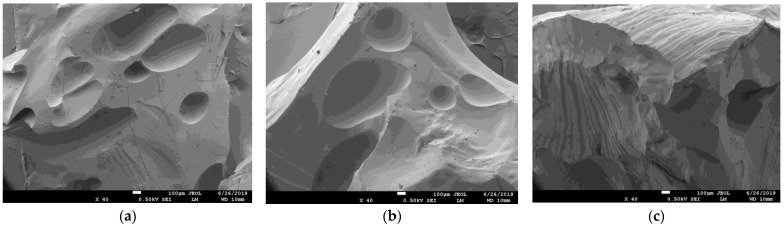
SEM photos of samples C with low content of NPK and LH (**a**), G with AM and LH (**b**), and H with LH (**c**) before swelling.

**Figure 5:**
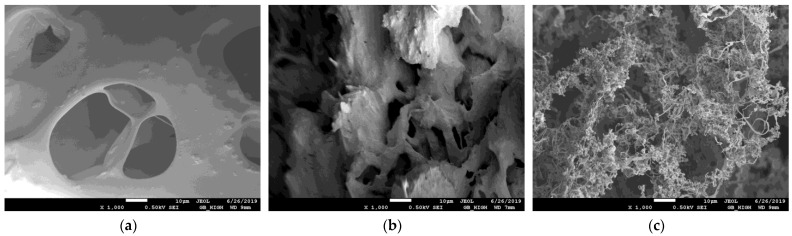
SEM photos of samples A with low content of NPK (**a**), B with high content of NPK (**b**), and C with low content of NPK and LH (**c**) after swelling and freeze-drying.

**Figure 6:**
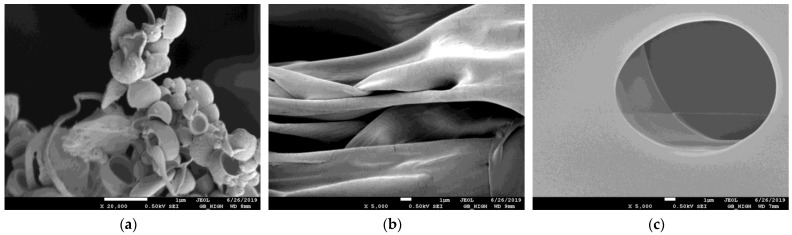
SEM photos of samples C with low content of NPK and LH in detail (**a**), G with AM and LH (**b**), and H with LH (**c**) after swelling and freeze-drying.

The surface morphology of superabsorbent polymers in lower magnification was not affected by the addition of NPK, LH, and AM (Figure 4). Prepared samples (before swelling) had similar characteristics. The surface heterogeneity caused by the increased amount of NPK incorporated into polymers was, in the lower magnitude, less visible (not shown). The addition of LH resulted in the brown coloration of the prepared samples. Although some authors (e.g., [53]) observed the increase in hydrogel porosity in the case of higher content of AM, the low AM amount added in polymers had no similar effect. In Figure 5, the comparison of swollen and freeze-dried hydrogels is shown. It was observed that the NPK addition resulted in a decrease in pore size. While the sizes of pores and voids in the hydrogel with low content of NPK were several tens of μm (Figure 5a), it decreased in magnitude if the addition of NPK was higher (Figure 5b). The surface of pores was covered by NPK fertilizer, similarly as in refs. [45,51]. The incorporation of LH in the superabsorbent polymer changed the structure of the swollen hydrogel. We can see that it is much miscellaneous and rich in different pores and voids of a wide range of their sizes. The detail of the structure formed by the combination of NPK and LH is shown in Figure 6a. This structure was detected only in the case of sample C; the higher content of NPK resulted in the collapse of this miscellaneous character, and the crystals originating from NPK covered the pore surface. Although the addition of AM was not too high in comparison with other studies [2,3,5,10,49,54,55,56], its presence in hydrogel significantly changed the shape of pores and voids from spherical to oblong slits. This observation can influence the swelling behavior of samples enriched with AM, as described in Section 2.3.

### 2.3. Water Absorbency

**Figure 7:**
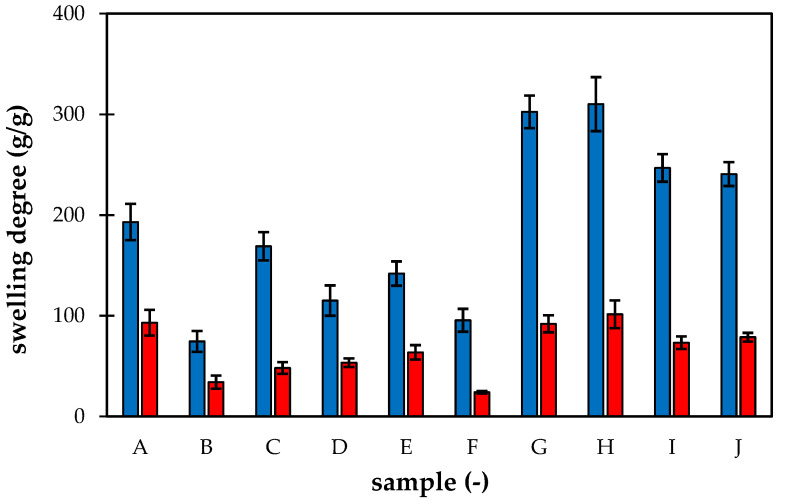
Swelling degree of prepared superabsorbent polymers in deionized (blue) and tap (red) water. Samples A and B contain only NPK, C, D, E, and F combination of NPK and LH, G and H only LH. Samples I and J are without nutrients. AM was added to samples E, F, G, and J.

The swelling degrees of superabsorbent polymers in deionized and tap water are shown in Figure 7. The values were determined on the basis of weight increase after 24 h. The swelling kinetics was studied in our previous work [13], and it was found that the swelling was relatively fast, and the equilibrium was achieved over several hours. Similar findings were published in refs. [3,5,10]. Dadhanyia et al. [5] observed maximum swelling degree after 20 h. Some superabsorbent polymers prepared by Raju et al. [3] achieved the maximum swelling degrees instantly, others in several minutes [57,58]. As can be seen, all prepared samples exhibited very good swelling properties. The samples differed from each other by their special compositions, as shown in Table 1. Higher contents of NPK in the structure of the sample induced a significant negative effect on swelling properties. The lowest values were obtained for samples containing higher amounts of NPK which caused the depression of swelling. Samples B, D, and F exhibited a much lower ability to absorb surrounding water. In contrast, samples with the addition of lignohumate swelled significantly better than samples without lignohumate. The addition of LH supported the absorption of water, which resulted in the greatest degree of swelling of samples G and H (without NPK) and an increase in swelling degree for samples containing a combination of NPK and LH (C, D, E, and F) in comparison with samples enriched by NPK alone LH (A and B). An adequate number of humic substances in the hydrogel polymeric network enhanced the hydrophilicity of superabsorbent samples. Free functional groups of humate (-OH, -COOH, -NH_2_, -SO_3_, quinonyl groups) interacted with acylamino and carboxyl groups of superabsorbent polymers and caused a collaborative absorbent effect. The positive effect of humic substances on swelling was observed in recent works [8,10,12,59,60].

Superabsorbents containing potassium or sodium humate [8,10,12] evinced both a higher swelling rate and a greater amount of absorbed water. However, some authors noted that the efficiency of humic addition to enhance swelling reached a maximum value that the addition of higher amounts of humic substances had no additional influence on swelling [12] or, conversely, began to have a negative influence [8,9,10,11]. A recommended allowance of humic substances differed between 3 and 30% wt. Chu et al. [9] showed that with the addition of a superabsorbent (1% wt.), water retention in soil increased 31 times and 40 times with the addition of a superabsorbent enriched with sodium humate. The increase in moisture in soil enriched by superabsorbent polymers was observed in recent work [61]. Our content of LH was in the lower limit. The increase in ionic strength usually suppressed the ability of the hydrogel to absorb water, and their degrees of swelling decreased in the presence of salts [10,11]. It corresponds with the increase in swelling degree in tap water observed for all prepared samples.

It was confirmed that the presence of acrylamide (samples E, F, and G) had little impact on the swelling ability of the studied hydrogels both in deionized and tap water. The samples without the presence of AM exhibited no lowering of the water absorbency. When we compare “pure” hydrogels differing only in the AM addition without NPK and LH (samples I and J), no difference in their swelling behaviors was observed. Some authors [53,62,63,64] studied the effect of AM content on the water absorbency and observed a maximum for different AM/AA ratios (0.1 [64], 0.4 [62], and 1 [53]). In this work, the AM addition is lower in comparison with published works [2,3,5,10] because of its toxicity [41,42,43,44]. The aim was to investigate potential positive effects on the prepared superabsorbent polymers, which were not confirmed in the case of water absorbency.

### 2.4. Rheological Properties of Prepared Superabsorbent Polymers

**Figure 8:**
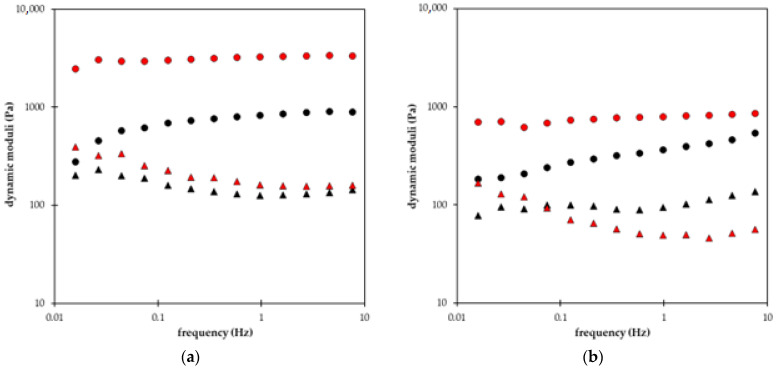

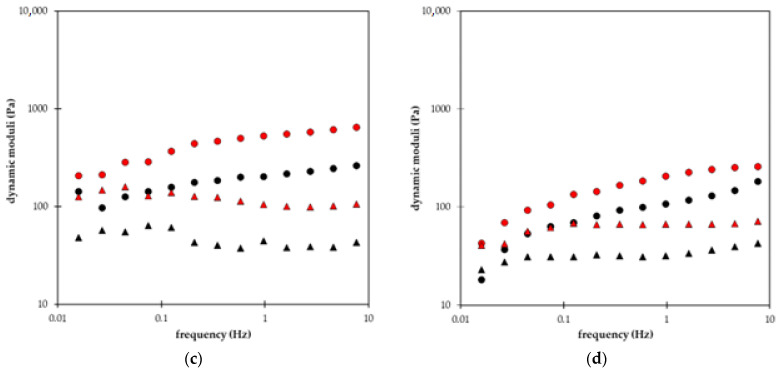
Storage modulus *G′* (circles) and loss modulus *G″* (triangles) in the dependence of frequency. Comparison of different NPK contents without LH (**a**): samples A (black) and B (red). Comparison of different NPK contents with LH (**b**): samples C (black) and D (red). Effect of AM addition to polymers containing NPK and LH (**c**): samples E (black) and F (red). Effect of AM addition to polymers containing LH without NPK (**d**): samples G (black) and H (red).

In Figure 8, the dynamic moduli of all superabsorbent polymers enriched by a fertilizer are compared. We can see that the storage modulus G′ proportional to the elastic component of the hydrogel is higher than loss modulus G″ proportional to the viscous component of the hydrogel. It means that all prepared materials have an elastic character. Storage modulus G′ increases with increasing frequency (samples A, C, E, F, G, and H) or seems to be practically independent of frequency (samples B and D). Samples B and D contain higher amounts of NPK, and no acrylamide was used in their preparation. When AM was added (sample F), the frequency dependence of G″ becomes increasing. A general effect of a higher amount of NPK is, therefore, the increase in (elastic) storage modulus (samples B, D, F). The increase in the difference between G′ and G″ for samples without AM and the decrease in (viscous) loss modulus G″ with increasing frequency supported the elastic character of prepared hydrogels (Figure 8a,b). Superabsorbent polymers containing lignohumate had lower dynamic moduli G′ and G″ (Figure 8b–d) in comparison with samples A and B enriched only by NPK (Figure 8a). The character of frequency dependence of loss modulus G″ changed with the LH addition. In contrast to Figure 8a, loss modulus increased slightly with increasing frequency or remained practically constant (except sample D with higher NPK content which kept decreasing G″ dependence). The lowest values of both moduli had samples G and H containing only lignohumate without NPK. The addition of acrylamide into superabsorbent polymers resulted in a stronger increase of storage modulus G′ with increasing frequency (compare samples D and F in Figure 8b,c) or in the decrease of both moduli in Figure 8d.

In summary, we can state several conclusions about how the individual constituents affect the viscoelastic behavior of superabsorbent polymers: (1) the increase in NPK content resulted in the increase in (elastic) storage modulus G′ and decreasing character of frequency dependence of (viscous) loss modulus G″; (2) the addition of LH caused the decrease in both moduli G′ and G″; (3) the addition AM resulted in the decrease in both moduli G′ and G″. It means that the NPK fertilizer supported the elastic character of prepared materials while the addition of lignohumate shifted the rheological behavior of superabsorbent polymers to more liquid. The more elastic character of samples with higher content of NPK means that they can resist mechanical stress, and their structure can be worse damaged. On the other hand, this effect is connected with the suppression of the release of lignohumate from superabsorbent D [13]. The addition of acrylamide did not improve the properties of prepared materials. The values of both moduli decreased with AM addition. The difference between G′ and G″ strongly decreased for samples with higher NPK content which resulted in a more liquid character of sample F (in comparison with sample D). The difference between both moduli for samples containing lignohumate (without NPK) remained practically the same (Figure 8d).

### 2.5. Stability of Prepared Superabsorbent Polymers

The stability of superabsorbent polymers was studied from three points of view. The first one was the time stability when the samples were repeatedly measured for one year. Other important aspects of the superabsorbents functionality in soil are drying/re-swelling cycles as well as the effect of freezing and defrosting of materials in winter.

**Figure 9:**
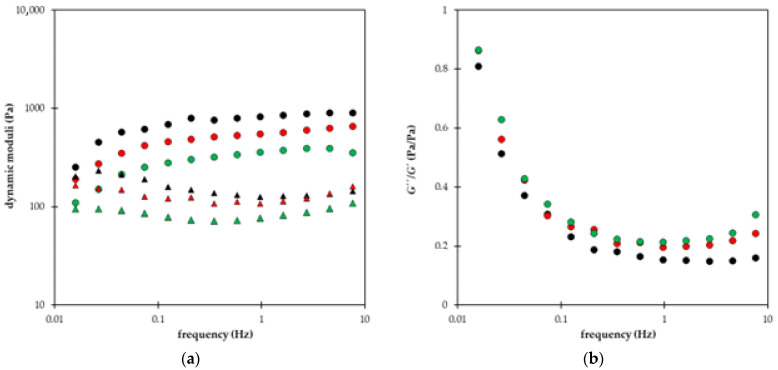
Time stability of sample A: Storage modulus *G′* (circles) and loss modulus *G″* (triangles) measured for sample A (**a**) and the ratio G″/G′ (**b**): after 24 h (black), 5 weeks (red), and 1 year (green).

An example of the time development of dynamic moduli measured for sample A is shown in Figure 9. We can see that both storage and loss moduli decreased gradually in time as materials lost their mechanical resistance. On the other hand, the decrease in both moduli was relatively favorable to the effect that the values did not differ in magnitude, and storage modulus remained above 100 Pa also after 1 year (Figure 9a). The ratio between loss and storage moduli increased slightly in time (Figure 9b). The G″/G′ ratio represents a criterion of the “liquid behavior” of the studied samples. When the ratio is equal to one, the storage and loss moduli have the same values, and the degrees of liquid and elastic extents are precisely balanced. Values higher than one denote that the sample behaves as a viscoelastic liquid.

**Figure 10:**
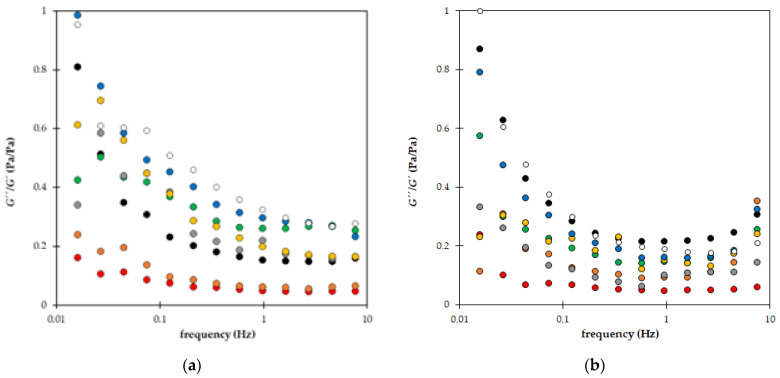
The ratio between loss and storage modulus after 24 h (**a**) and 1 year (**b**): samples A (black), B (red), C (green), D (orange), E (grey), F (yellow), G (blue), and H (white).

**Figure 11:**
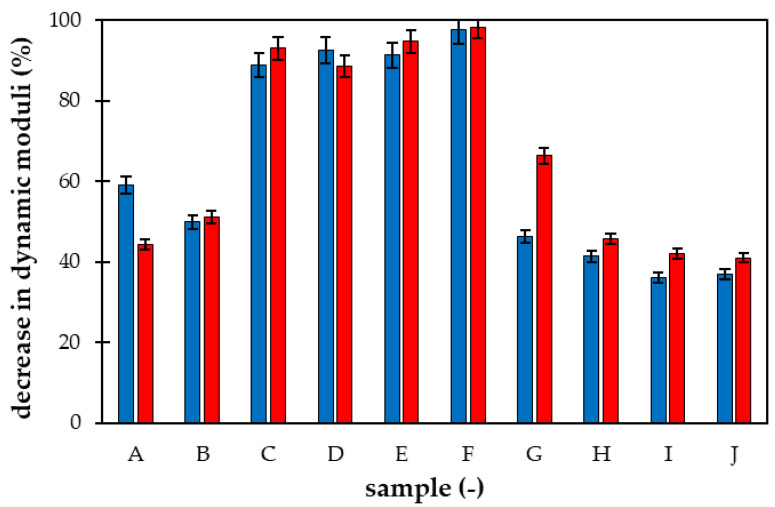
The average decrease in storage modulus (blue) and loss modulus (red) after 1 year. Samples A and B contain only NPK, C, D, E, and F combination of NPK and LH, G and H only LH. Samples I and J are without nutrients. AM was added to samples E, F, G, and J.

The time stability of superabsorbent materials enriched by nutrients (NPK and LH) is compared in Figure 10. The ratio between loss and storage moduli after 24 h and 1 year are compared. The trend of a slight increase in the G″/G′ ratio observed for sample A (Figure 9b) was not confirmed for all prepared materials. In some cases (samples D, E, F, G, and partially H), the ratio decreased after a year, which means that the participation of viscous character decreased. It is not caused by a strengthening of hydrogel structure but by the different rates of decrease in individual moduli, as shown in Figure 11. As can be seen, the highest decrease in both moduli was observed for samples containing NPK in combination with LH. The decrease for these samples (C, D, E, and F) was between 80 and 90%. In contrast, the decrease in moduli obtained for samples enriched with only one type of fertilizer (NPK or LH) was lower and comparable (samples A, B, G, and H). The effect of AM addition on the decrease in moduli was negligible.

**Figure 12:**
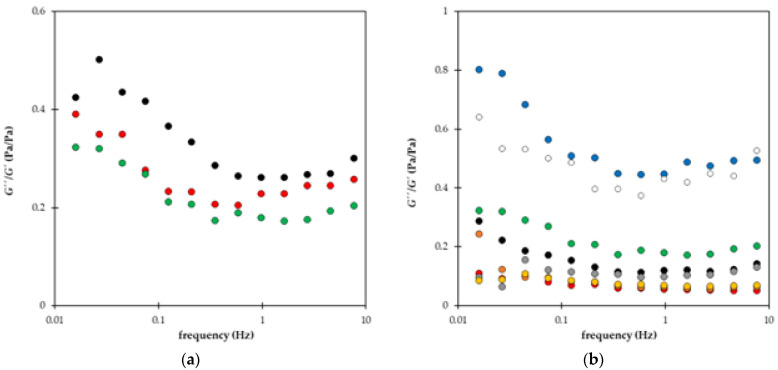
The ratio between loss and storage modulus after one (black), three (red), and five (green) re-swelling cycles for sample C (**a**). The *G″*/*G′* ratio after five cycles (**b**): samples A (black), B (red), C (green), D (orange), E (grey), F (yellow), G (blue), and H (white).

Changes in the rheological behavior of superabsorbent polymers during repeated swelling are shown in Figure 12. We can see that the G″/G′ ratio decreased with the number of cycles (Figure 12a). In Figure 12a, the examples of data obtained for sample C are shown. A similar effect of re-swelling on the rheological behavior was observed for all prepared hydrogels. The viscous extent decreased gradually with a number of cycles. The results obtained for individual samples differed in the final G″/G′ ratio (Figure 12b). The highest extent of liquid character was determined for samples G and H containing only lignohumate without NPK. Samples containing NPK evinced less viscous participation and seemed to be more resistant to mechanical stresses. The gradual decrease during re-swelling cycles is caused by the increase in both dynamic moduli, which was observed for all prepared hydrogels, e.g., the storage modulus of samples E and F increased after the fifth cycle more than three times. The reinforcement of prepared materials during re-swelling cycles is connected with the release of nutrients from their structure described in some refs. [13,45,46,47,51,65].

**Figure 13:**
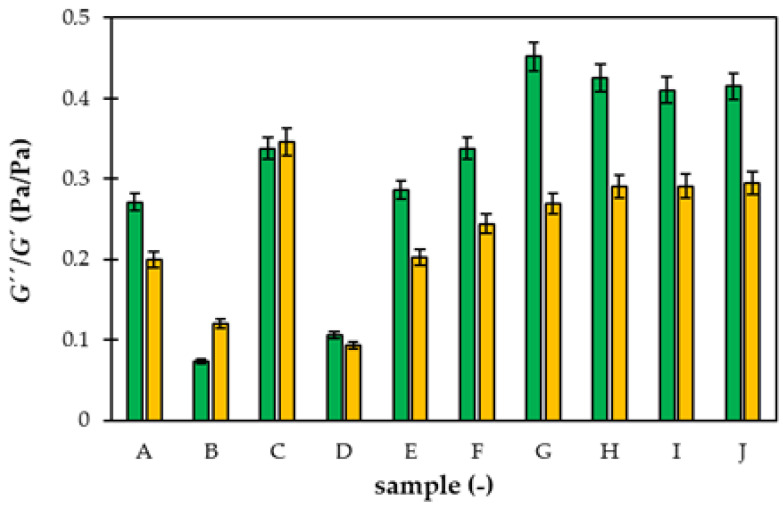
The average *G″*/*G′* ratio before (green) and after (yellow) freezing. Samples A and B contain only NPK, C, D, E, and F combination of NPK and LH, G and H only LH. Samples I and J are without nutrients. AM was added to samples E, F, G, and J.

The effects of freezing on the rheological character of superabsorbent polymers are summarized in Figure 13. In general, the freezing and defrosting resulted in a decrease in the G″/G′ ratio (except samples B and C). It means that the portion of liquid character decreased, and samples became more resistant to mechanical stresses. In contrast, the behavior of sample B shifted to a more liquid character. In this case, the reinforcement by a higher content of NPK disappeared during freezing. The ratio G″/G′ remained approximately constant for samples C and D, combining the additions of NPK and lignohumate, but its value decreased if AM was added in the superabsorbent structure (samples E and F). The biggest decrease in the G″/G′ ratio was observed for samples G and H containing LH without NPK. The addition of AM had a negligible effect on pure superabsorbents (samples I and J).

## 3. Conclusions

Ten superabsorbent polymers based on polyacrylic acid were prepared and investigated from the point of view of their potential use in soil as a water reservoir and source of mineral and organic nutrients. The release of nutrients and the effect on plant growth were studied in detail in our previous work [13]. The aim of this work was to consider the behavior and stability of prepared materials, including repeated swelling/drying cycles and the freezing/defrosting process. The addition of NPK had a negative effect on the ability of polymers to absorb water, while the lignohumate supported the superabsorbent swelling. SEM analysis showed that the lignohumate supported the formation of complex structures with different pores and voids. The time stability was studied in one year. Both dynamic moduli decreased in time, but their ratio often remained practically the same. The highest decrease was observed for materials combining the NPK and lignohumate addition. Repeated swelling/drying cycles resulted in a decrease in the *G″*/*G′* ratio. It means that the character of materials that shifted to a more elastic and liquid character gradually decreased, which was connected with the gradual release of fertilizers. The more liquid character remained only for samples containing lignohumate without NPK. In general, the freezing and defrosting of prepared materials caused a decrease in their liquid character. Samples combined with a higher amount of NPK became more liquid, and the character of samples combining NPK with lignohumate (without acrylamide) remained the same. This was the one case where the addition of acrylamide resulted in the change of sample properties (compare samples C and D with E and F). Its effect on the pure superabsorbent polymers (without fertilizers) was negligible, and a positive effect on the utility properties of prepared materials was not confirmed.

## 4. Materials and Methods

### 4.1. Chemicals

Ten different samples of superabsorbent polymers were synthesized through the rapid solution polymerization of partially neutralized acrylic acid under normal atmospheric conditions. Some samples were based on acrylic acid and acrylamide mixture. Powders of potassium lignohumate (Amagro s. r. o., Prague, Czech Republic) and NPK 20-8-8 (Lovochemie a.s., Lovosice, Czech Republic) were used as organic and mineral nutrients.

Acrylic acid, acrylamide, *N,N*'-methylenebisacrylamide, and potassium peroxydisulfate were purchased from Sigma-Aldrich (St. Luis, MO, USA). KOH was purchased from Penta (Prague, Czech Republic). NPK 20-8-8 was purchased from Lovochemie a.s. (Lovosice, Czech Republic). Lignohumate was kindly provided by Amagro (Prague, Czech Republic). Its main characteristics, such as elemental composition and structural features, can be found in refs. [26,28,34,40].

### 4.2. Preparation of Superabsorbent Polymers

The basic materials were prepared using the following method. The weight quantity of AA (57 g) was dissolved in distilled water (100 cm^3^). The AA solution (25 cm^3^) was neutralized by 10 cm^3^ of 8.5 M potassium hydroxide solution and crosslinked by MBA (0.016 g). Then, the initiator KPS was added (0.5 g). The mixture was continuously heated and stirred until reaching the temperature of approximately 85 °C; then, the highly viscous mixture was removed from the beaker and placed in an oven for 24 h, which was settled at 80 °C. The dried product was crushed by a hammer into small pieces [9,13]. When samples with NPK (A, B), LH (E, F), and its combination (C, D) were prepared), their powder was added to the AA solution neutralized by KOH. Alternatively, AM (0.75 g in 1.5 cm^3^ of distilled water) was mixed with AA before neutralization (E, F, G, and J). The compositions of prepared samples are described in detail in Table 1.

### 4.3. Characterization of Superabsorbent Polymers

Prepared superabsorbent polymers were characterized by several methods: FT-IR spectrometry, scanning electron microscopy (SEM), and water absorbency.

FT-IR spectra of lignohumate, NPK, and prepared superabsorbent polymers were measured by ATR technique over the range of 4000–400 cm^−1^. FT-IR spectrometer (Nicolet iS 50) operating with a peak resolution of 4 cm^−1^ and 128 scans were performed on each acquisition. All prepared samples were measured in a dry state before swelling.

Scanning electron microscopy (JOEL JSM-7600F, Thermo Fisher Scientific, Waltham, MA, USA) was used for the characterization of the surface morphology of prepared superabsorbent polymers before swelling and then for the characterization of their pore structure in swollen forms after freeze-drying.

Water absorbency was determined by means of swelling experiments in deionized and tap water. Superabsorbent polymers in the form of xerogel were mixed with water in the ratio of 50 mg: 100 cm^3^. The swelling degree was determined on the basis of weight increase after 24 h [13]. The weight increase caused by the absorption of water was expressed as the so-called swelling degree *Q*. It was calculated as the amount of absorbed water (expressed as the difference between the weight of hydrogel *m_h_* and the weight of xerogel *m_x_*) normalized on the weight of xerogel [3,4,5,8,10,12,13,54]:

(1) $$Q=\frac{m_{h}-m_{x}}{m_{x}}.$$

### 4.4. Rheological Properties of Prepared Superabsorbent Polymers

Rheological properties were determined by means of an Anton Paar Physica MCR 501 rheometer (Anton Paar, Gray, Austria), partially according to a previously reported method [55,56]. The measurement was performed using a parallel plate system (PP25-SN6375, 25 mm diameter) with a 1 mm gap. First, a conditioning step was performed (3 min), followed by a strain sweep test (strain: 0.01–100%, 10 rad/s) to determine the linear viscoelastic region. The region ranged from 0.01 to 1% for almost all samples, except E and G. These latter two were more pliable; their linear viscoelastic region ranged from 0.01 to 0.5%. Therefore, an amplitude of deformation of 0.1% was chosen as suitable for all further experiments (frequency sweeps).

Frequency sweep measurements were taken for all samples under the following conditions: strain, 0.1%, and frequency, 0.1–628 rad/s. Conditioning steps were performed before each measurement. Viscoelastic measurements in oscillatory shear flow—frequency sweep and strain sweep—were performed for each sample, and the obtained values of moduli *G′* and *G″* were compared. The storage modulus G′ is proportional to the extent of the elastic component, and the loss modulus G″ is proportional to the extent of the viscous component of the system. The strength of prepared samples can be characterized in summary by means of their ratio *G″*/*G′*. Three repetitions of viscoelastic measurements were performed for each sample, and the obtained values of moduli were checked for reproducibility. Three repetitions of viscoelastic measurements were performed for each sample, and the obtained values of moduli were checked for reproducibility.

### 4.5. Stability of Prepared Superabsorbent Polymers

The first stability study was based on the repeat measurement of rheological properties over time (up to one year). Samples were kept in a swollen state; they were stored in a desiccator with water to prevent drying out.

The next experiment was focused on the re-swelling process on superabsorbent polymers. It means that superabsorbent polymers were repeatedly dried and re-swollen in order to investigate potential changes in their properties in the cycles. The cycles were realized with deionized water as described above (Section 4.3).

Since the superabsorbent polymers should be used outside in soil, other characteristics were focused on their freezing and defrosting. Samples were frozen in their swollen form (after 1st cycle) at −19 °C (24 h) and then defrosted at laboratory temperature (24 h). Viscoelastic moduli (*G′* and *G″*) were measured after each cycle of re-swelling as well as after the defrosting of samples.

## Figures and Tables

**Table 1:** The composition of prepared superabsorbent materials.

Sample	A	B	C	D	E	F	G	H	I	J
AM (g)	0	0	0	0	0.75	0.75	0.75	0	0	0.75
NPK (g)	0.660	6.602	0.660	6.602	0.660	6.602	0	0	0	0
LH (g)	0	0	1	1	1	1	1	1	0	0

## Data Availability

On request.
